# On the relationship between mind perception and social support of chatbots

**DOI:** 10.3389/fpsyg.2024.1282036

**Published:** 2024-03-06

**Authors:** Inju Lee, Sowon Hahn

**Affiliations:** Human Factors Psychology Lab, Department of Psychology, Seoul National University, Seoul, Republic of Korea

**Keywords:** chatbot, social support, mind perception, human-like mind, user experience, human-computer interaction (HCI)

## Abstract

The social support provided by chatbots is typically designed to mimic the way humans support others. However, individuals have more conflicting attitudes toward chatbots providing emotional support (e.g., empathy and encouragement) compared to informational support (e.g., useful information and advice). This difference may be related to whether individuals associate a certain type of support with the realm of the human mind and whether they attribute human-like minds to chatbots. In the present study, we investigated whether perceiving human-like minds in chatbots affects users’ acceptance of various support provided by the chatbot. In the experiment, the chatbot posed questions about participants’ interpersonal stress events, prompting them to write down their stressful experiences. Depending on the experimental condition, the chatbot provided two kinds of social support: informational support or emotional support. Our results showed that when participants explicitly perceived a human-like mind in the chatbot, they considered the support to be more helpful in resolving stressful events. The relationship between implicit mind perception and perceived message effectiveness differed depending on the type of support. More specifically, if participants did not implicitly attribute a human-like mind to the chatbot, emotional support undermined the effectiveness of the message, whereas informational support did not. The present findings suggest that users’ mind perception is essential for understanding the user experience of chatbot social support. Our findings imply that informational support can be trusted when building social support chatbots. In contrast, the effectiveness of emotional support depends on the users implicitly giving the chatbot a human-like mind.

## 1 Introduction

We face many stressful events in our lives and tend to share them with others who provide various support including helpful advice or empathy. All valuable resources for dealing with stress events provided through social interactions and relationships are referred to as *social support* (Lin et al., 1979; Hobfoll, 1988; Cohen, 2004). Social support is divided into several subtypes based on resource characteristics, including informational support and emotional support (Cobb, 1976; House et al., 1985; Cohen, 2004). *Informational support* refers to the provision of useful advice or information that helps resolve stressful events, while *emotional support* refers to expressing empathy, encouragement, and care, making individuals feel loved and valued by the support provider. Social support promotes individuals’ psychological well-being by encouraging them to handle their stressful events in more successful ways (Cohen and Wills, 1985; Cohen, 2004).

Chatbots, artificial intelligent (AI) agents that interact with users through written texts (Rapp et al., 2021), can be another source of social support. Social chatbots, or chatbots that perform social functions, are designed to provide users with social support while having daily conversations and building social relationships with them (Shum et al., 2018; Ta et al., 2020). Mental health care chatbots are built to offer a variety of social support to help users deal specifically with their mental health problems, such as depression and anxiety disorders (e.g., Fitzpatrick et al., 2017; Inkster et al., 2018). Previous studies have shown that social support from chatbots can improve psychological health (Fitzpatrick et al., 2017; Ly et al., 2017; Inkster et al., 2018; Mehta et al., 2021; Meng and Dai, 2021).

The social support provided by chatbots is usually designed to mimic that provided by humans. When individuals share stressful events, the chatbot not only provides useful information but also sends messages expressing empathy, as humans normally do (e.g., Fitzpatrick et al., 2017; Inkster et al., 2018; Ta et al., 2020). However, human-like social support from chatbots does not always guarantee positive user responses. Previous research showed users have conflicting attitudes toward a particular type of social support—emotional support. Some users acknowledge and appreciate chatbots’ empathic expression, whereas others feel discomfort when the chatbot expresses emotional support (e.g., Liu and Sundar, 2018; Urakami et al., 2019; Bae Brandtzæg et al., 2021). Better understanding of users’ opposing attitudes is needed to optimize the effectiveness of chatbot support.

In the present study, we explored the conflicting user experience of chatbot support, considering the characteristics of support, especially whether it is associated with the realm of the human mind. When an AI agent appears to have the abilities that they ultimately lack, people may experience feelings of discomfort (Gray and Wegner, 2012). People generally consider emotion as unique to humans (Gray and Wegner, 2012) and perceive AI agents as lacking it (Gray et al., 2007; Jacobs et al., 2022). People may expect the emotional support provider to have and feel emotions; therefore, they may reject emotional support from chatbots because chatbots do not have such capabilities. Individuals can only acknowledge the ability of chatbots to provide emotional support when they are perceived to have a human-like mind. On the contrary, people may accept informational support from chatbots even if they do not humanize them. Any computer systems, including chatbots, are expected to be proficient in searching and providing useful information, as confirmed in previous studies (Brandtzaeg and Følstad, 2017; Kim et al., 2018).

Although previous studies have investigated the importance of mind perception in understanding chatbots’ social support (Liu and Sundar, 2018; Urakami et al., 2019; Bae Brandtzæg et al., 2021), none have directly measured users’ mind perception of a specific chatbot and examined the relationship between this perception and various types of social support. In the current study, we examined whether explicit or implicit mind perception is related to users’ attitudes toward chatbots’ social support. Additionally, we explored whether the strength of the relation differs depending on the type of chatbot support.

Our research makes several contributions. First, it provides empirical evidence that users’ acceptance of chatbots’ support differs depending on whether they explicitly perceive human-like minds in the chatbots. The importance of implicit mind perception varies depending on specific types of social support. Specifically, the relation between implicit mind perception and user attitude toward chatbots’ support is stronger when the chatbots provide a type of support that requires a human-like mind (i.e., emotional support). Our findings imply that users’ implicit mind perception should be considered to enhance the positive effects of support when designing chatbots for emotional support. In contrast, chatbots’ informational support is more reliable in inducing positive user reactions.

## 2 Background

### 2.1 Mind perception and its effect on human-chatbot interactions

Whether a chatbot is considered a mindful entity depends on the observers’ perception. In other words, for the same chatbot, some people may believe it has its own mind, while others may not (e.g., Lee et al., 2020). In addition, their perception can change depending on the context. For example, people are more likely to attribute humanness to AI agents when they have a more human-like appearance (Disalvo et al., 2002).

Individuals can attribute human-like minds to chatbots through implicit and explicit processes. *Implicit processes* are automatic/spontaneous and made without awareness, whereas *explicit processes* are controlled and made with awareness (Nosek, 2007; Low and Perner, 2012). Chatbots are computers and do not fundamentally have human-like properties. Nevertheless, sometimes people implicitly mentalize them while explicitly recognizing that they lack human-like minds. Reeves and Nass (1996) demonstrated that people exhibited social responses to computers and treated them as if they were human beings, even when they explicitly acknowledged that computers are not humans. Additionally, previous studies have shown that explicit and implicit mentalizing do not always go together (Banks, 2020, 2021).

Implicit mentalizing has been explored within the computers are social actors (CASA) paradigm. This paradigm assumes that, when computers or machines display enough social cues to users, users instinctively treat them as though they are humans (Nass et al., 1994; Nass and Moon, 2000). Implicit attribution of mindfulness to computers elicits similar responses from users as when they interact with other people. For example, people tend to assess a particular computer’s performance more positively when they are asked to answer an evaluation questionnaire on the same computer compared to independent sources (e.g., a paper questionnaire or a different computer), which implicates that people perceive it as a direct evaluation of the computer, leading them to show politeness to the computer (Nass et al., 1994). People also apply gender stereotypes to computers while automatically treating them like human beings (Nass et al., 1994; Nass and Moon, 2000). Therefore, based on the CASA paradigm, chatbots’ human-like social support is expected to have a positive effect on human-chatbot interactions similar to how support from humans does in human-human interactions. Previous studies have shown that users perceive the empathic expression of computers positively when these agents display sufficient social cues (Bickmore and Picard, 2004; Brave et al., 2005).

Explicit mentalizing has been investigated through theories of mind perception. Theories of mind perception explain that people perceive the mind of a particular entity in dimensions of agency and experience (Gray et al., 2007; Gray and Wegner, 2012). *Agency* is the ability to think and act volitionally, whereas *experience* is the ability to feel sensations and emotions. Researchers have explored the general perception of various entities and found that adult humans are perceived as having both high agency and experience, whereas AI agents, including chatbots, are perceived as having a low-middle level of agency but lacking experience (Gray et al., 2007; Jacobs et al., 2022). The higher the rating of an entity’s agency and experience, the more human-like people perceive it to be. The perception of AI agents can change through anthropomorphism, which is the process of attributing human-like characteristics (e.g., thought, intention, desire, and emotion) to nonhuman entities (Epley et al., 2007). The perceived agency and experience of the AI agent can increase when people anthropomorphize the agent (Yam et al., 2021).

Explicitly attributing human-like minds to AI agents usually has a positive effect on the interactions between humans and the agents. For example, Lee et al. (2020) found that mind perception in a chatbot increased co-presence and closeness with the chatbot. Yam et al. (2021) showed that when users anthropomorphized a service robot, they perceived higher agency and experience in the robot, which enhanced their overall satisfaction with the hotel where the robot worked. In addition, when users perceive an intelligent personal assistant to sense, think, and act autonomously (i.e., ascribe mind attributes to the agent), they perceive the agent as more competent, which leads to a higher intention to continuously use the agent (Hu et al., 2021).

**Figure 1:**
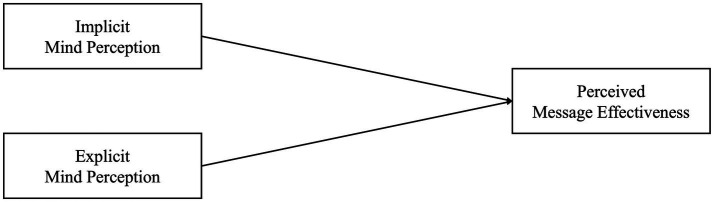
Conceptual framework for hypotheses 1–1 and 1–2.

Based on previous research, we expected implicit and explicit mind perception to be related to a more positive user attitude toward chatbots’ social support. We examined perceived message effectiveness (i.e., the extent to which users perceive chatbots’ messages as effective) for user attitude in the context of chatbots providing social support, as previous research did (Liu and Sundar, 2018). Furthermore, we measured mind perception as a variable rather than manipulating it because users’ mind perception is influenced by a variety of factors, making it challenging to manipulate, even when incorporating anthropomorphic cues in chatbots (Epley et al., 2007; Waytz et al., 2010). The corresponding framework is illustrated in Figure 1 and the corresponding hypotheses were as follows:

*Hypothesis 1-1*: When users implicitly perceive human-like minds in chatbots, they perceive the chatbots’ messages as more effective.

*Hypothesis 1-2*: When users explicitly perceive human-like minds in chatbots, they perceive the chatbots’ messages as more effective.

### 2.2 Importance of mind perception depending on the type of social support

Theories of mind perception explain how the perception of entities’ minds is related to the way people perceive their behaviors and interact with them. For example, when an agent is perceived as having agency, it is attributed with the responsibility for its behavior (Waytz et al., 2010). Additionally, when individuals dehumanize other people, or deny others’ human mind abilities, they tend to have more negative attitudes toward others (Hodson and Costello, 2007; Esses et al., 2008).

When AI agents behave as though they have mental abilities that they essentially lack, people have a negative impression toward them. In general, people assume that experience is fundamental to human beings (Gray and Wegner, 2012) and perceive AI agents as lacking it (Gray et al., 2007; Jacobs et al., 2022). Consequently, people feel uncanniness when AI agents appear to have experience (Gray and Wegner, 2012). Relatedly, Stein and Ohler (2017) showed that people perceived the empathetic and emotional expression of an AI agent more negatively when they acted as though they had mental abilities, and suggested that it was because people might not expect them to have those capabilities.

The concept of mind perception may explain why conflicting user attitudes frequently occurred for chatbots’ emotional support compared to informational support. Different types of social support offer different recourses (Cobb, 1976; House et al., 1985; Cohen, 2004); therefore, what mental abilities the support provider is expected to possess can vary depending on the type of support provided. On the one hand, informational support abilities, such as understanding stress situations and exploring useful information to manage these, are related to agency. On the other hand, emotional support abilities, such as understanding stress situations and the thoughts and feelings of support-seekers, are related to agency, but also include sharing similar feelings with support-seekers and feeling a desire to comfort them, which are related to experience.

The importance of attributing human-like minds therefore may vary depending on the type of support provided by chatbots. Chatbots are perceived as having agency to some extent, but lacking experience (Gray et al., 2007; Jacobs et al., 2022). Individuals might expect chatbots to provide informational support and acknowledge it, even though they do not ascribe human-like minds to chatbots. Previous studies have shown that users usually expect a chatbot to perform informational analysis and retrieval (Brandtzaeg and Følstad, 2017; Kim et al., 2018). On the contrary, people consider chatbots’ emotional support as artificial and less genuine unless they humanize and attribute experience abilities to the chatbot. In a previous study, users denied an AI agent’s empathetic expressions while considering that it pretended to understand their emotions (Urakami et al., 2019). However, users who appreciated chatbots’ empathic expressions reported feeling as if they were talking to a human (Fitzpatrick et al., 2017; Bae Brandtzæg et al., 2021), which suggests the importance of mentalizing chatbots for accepting their empathy.

Implicit attribution of a human-like mind may be sufficient to evoke a positive experience from a chatbot’s emotional support, even when individuals do not explicitly mentalize them. As previously mentioned, the CASA paradigm, which explains positive user experiences of chatbots’ emotional support, assumes that users automatically attribute humanness to computers (Nass et al., 1994; Nass and Moon, 2000).

**Figure 2:**
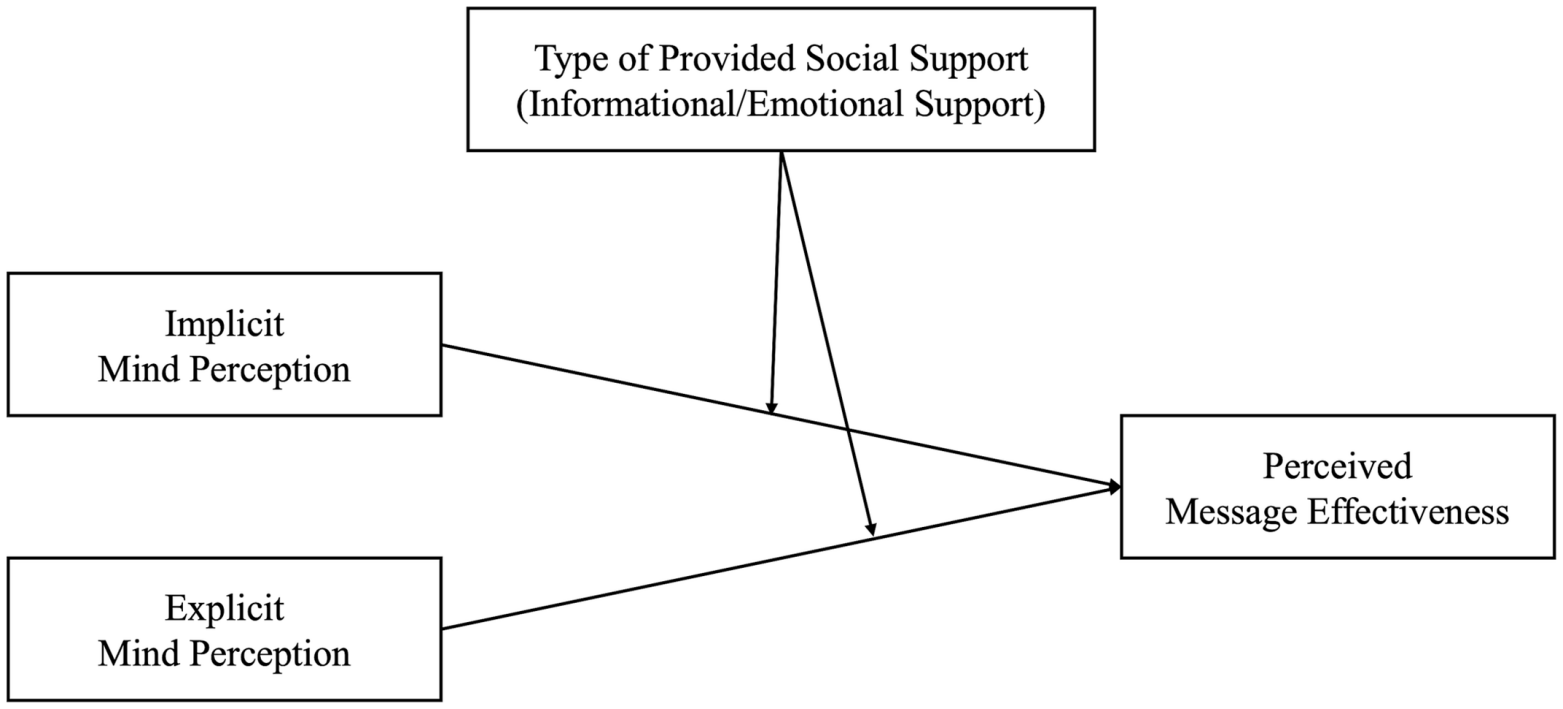
Conceptual framework for hypotheses 2–1 and 2–2.

Taken together, we hypothesized that the necessity of perceiving human-like minds in chatbots for inducing positive user reactions varies depending on the type of support provided by the chatbot. Attributing human-like minds might be more crucial for emotional support to elicit desirable effects than informational support. As we did for Hypotheses 1–1 and 1–2, we measured perceived message effectiveness for user attitude toward chatbots’ social support. The conceptual framework is illustrated in Figure 2 and the corresponding hypotheses were as follows:

*Hypothesis 2-1*: The relationship between implicit mind perception and perceived message effectiveness is stronger when the chatbots provide emotional support compared to informational support.

*Hypothesis 2-2*: The relationship between explicit mind perception and perceived message effectiveness is stronger when the chatbots provide emotional support compared to informational support.

## 3 Materials and methods

### 3.1 Participants

A total of 163 individuals participated in this study. We recruited participants using online recruitment systems and communities and rewarded them with either course credits, gift vouchers, or payments. Twenty-six individuals were excluded from the analysis owing to incomplete data. The final sample comprised 137 participants (56.2% female). The mean age was 23.3 (SD = 6.1), ranging from 18 to 49 years. All the participants were Korean and 43.1% had experience using chatbots. We conducted retrospective power analyses (Faul et al., 2009) with our sample size at α =0.05. The current study had sufficient power (>87%) to detect mid-to-large effects (Cohen’s *d* = 0.65 or partial η^2^ = 0.10). The power to detect medium effects (Cohen’s *d* = 0.50 or partial η^2^ = 0.06) was as follows: 81% for *t*-tests and 65% for two-way analysis of variance (ANOVA) tests.

### 3.2 Experiment design

We used a between-subjects experimental design, and the type of social support provided by the chatbot was manipulated as follows: (1) base condition, (2) informational support condition, (3) emotional support condition. In the base condition, the chatbot prompted participants to discuss their interpersonal stress events by asking them several questions. Specifically, participants were asked what the stressful situation was about, what thoughts and emotions arose from the experience, how they behaved in the situation, how they would change their behavior, and how their emotions changed after conversing about the stressful event. It would function as a form of social support since previous research showed that, when chatbots questioned what type of person the user was and what thoughts, feelings, and beliefs the user had, these questions facilitated introspection and encouraged users to become aware and understand themselves better (e.g., Ta et al., 2020; Bae Brandtzæg et al., 2021). In the informational or emotional support conditions, the chatbot additionally provided the corresponding social support, as follows. For informational support, the chatbot provided relationship advice, according to the phase of the interpersonal relationship: (1) building, (2) maintaining, and (3) ending. We created the chatbot’s advice by referring to the *Psychology of Human Relationships* (Kwon, 2017). For emotional support, the chatbot expressed phrases that conveyed understanding toward participants’ thoughts, emotions, and behaviors. It also expressed encouragement to participants. We adapted the emotional support content from Meng and Dai (2021). Participants were randomly assigned to one of three conditions (42 in the base condition, 48 in the informational support condition, and 47 in the emotional support condition).

### 3.3 Experiment chatbot

To control the flow of conversations between a chatbot and the participants, we instructed the chatbot to lead the conversation according to predefined scripts. We built a script-based chatbot using Chatfuel^1^ and integrated it into Facebook Messenger. Following predefined scenarios, our chatbot sent various messages that facilitated social interactions with participants and dealt with interpersonal stressful events.

To encourage participants to anthropomorphize and attribute a human-like mind to our chatbot, we utilized several anthropomorphic and social cues suggested by previous studies (Gong, 2008; Araujo, 2018; Go and Sundar, 2019; Schuetzler et al., 2020; Adam et al., 2021; Schanke et al., 2021). Those cues facilitate anthropomorphism by increasing an agent’s social presence (i.e., the degree to which an agent is salient in the interaction; Short et al., 1976) and signaling its identity. First, we tried to make our chatbot’s messages more human-like and personalized by sending responses that reflected the contents of participants’ previous messages. We integrated the Dialogflow^2^ AI system into our chatbot and trained it to predict the types of relationships in which the participants had stressful events (e.g., father, mother, friends, and romantic relationship) and the types of emotions experienced by the participants (e.g., sad, depressed, and stressed). The chatbot sent messages that reflected the predicted types of relationships or emotions.

We also used various other methods to attribute human-likeness to the chatbot. For instance, by expressing the same meaning in various forms of messages (e.g., Okay, Ok, I see). To make an impression that our chatbot typed messages in real-time, we added some delay (1 s ~ 6 s depending on the length of messages) and showed typing indicators (i.e., three dots) before sending messages. In addition, we used a human-like image and name (Allen) for the chatbot. Lastly, our chatbot presented its identity using first-person singular pronouns.

In addition to imbuing human-likeness, to facilitate anthropomorphism, we tried to elicit social responses and reduce social distances by making the chatbot say “hi” and “goodbye” to participants and engage in small talk (i.e., asking about participants’ experience of chatbot usage).

### 3.4 Measurements

#### 3.4.1 Perceived message effectiveness

To measure the extent to which participants perceived the chatbot messages as effective, we used four items from Holmstrom et al. (2005) after translating them into Korean. The items were originally used for measuring “the perceived effectiveness of the helper’s behavior” in Holmstrom et al.’s (2005) study; they were also used to examine chatbot message effectiveness (Liu and Sundar, 2018). Participants were asked to rate each item on a 7-point Likert scale. Cronbach’s α was 0.86. The mean and standard deviation were 4.4 and 1.3, respectively. Example items included: “ineffective” – “effective” and “helpful” – “unhelpful.”

#### 3.4.2 Explicit mind perception in a chatbot

To assess the extent to which participants explicitly perceived a human-like mind in the chatbot, we utilized items from Lee et al. (2020) and Gray and Wegner (2012) after translating them into Korean. We used two items from Lee et al. (2020) that assessed the perception of the chatbot’s ability to think and behave, which operationalized the capacity related to agency (Gray et al., 2007), and two items from Gray and Wegner (2012) to assess the perception of the chatbot’s ability to feel pain and fear, which operationalized the capacity related to experience (Gray et al., 2007). Participants were asked to answer each item on a 7-point Likert scale. Cronbach’s α was 0.87. The mean and standard deviation were 2.9 and 1.3, respectively. Example items included: “I felt that Allen was able to think by itself” and “I felt that Allen had the capacity to feel pain.”

#### 3.4.3 Implicit mind perception in a chatbot

We adapted one task from Banks (2020) to examine whether participants implicitly perceived a human-like mind in the chatbot. The Banks’s (2020) tasks measure users’ implicit mind perception toward a robot. Since chatbots have different unique characteristics from robots (e.g., no voice and physical body), only one task was determined valid to be utilized in the context of interactions between our chatbot and a human: the white lie scenario. In that scenario, the AI agent does not have to possess a voice or physical body but the ability to converse, which makes the scenario appropriate to be utilized for a chatbot.

In our measurement, the participants were presented with a cartoon of the white lie scenario. In the scenario, our chatbot received a gift from a human; however, it was not the gift that it wanted to have. Nevertheless, the chatbot answered that it liked the gift when the human asked about it. After reading the cartoon, the participants were asked to judge whether the chatbot lied and write down their explanation of why the chatbot said that it liked the gift.

Based on Banks’s (2020) codebook, two raters analyzed the participants’ answers about the reasons for the chatbot’s behavior and coded the presence of implicit mind attribution. More specifically, the raters examined whether the participants indicated an internal mental state as the reason for that behavior. Internal mental state refers to “having any kind of thought, feeling, motivation, or condition that suggests the agent was actively thinking about how to respond, deciding how to react, driven to behave in a certain way, or emotionally moved to behave in that way” (Banks, 2020, p.2 in the codebook). If the answer contained an indication of the chatbot’s internal mental state, the answer was coded as 1, otherwise 0. For example, an answer such as “the chatbot might *not want to hurt* the gift giver’s feelings” was coded as 1, while one like “the *algorithm behind the chatbot* made it respond in that way” was coded as 0. We calculated the Cohen’s kappa score to measure intercoder reliability and the score was 0.86, implying enough agreement. Eighty-three participants showed indicators of mentalizing in their answers while 54 did not.

### 3.5 Procedure

The study procedure was approved by our university’s institutional review board (IRB No. 2109/002–031). The experiment was conducted online. Participants were asked to participate in this study through their mobile devices because they had to converse with our chatbot through the Facebook Messenger application. After signing up for the study, participants received an online study link. The first page provided participants with a written description of the study. Only those who consented to participate were included in the study.

Participants were informed of the guidelines for chatting with our chatbot and given another link directing them to a conversation with the chatbot on the Facebook Messenger application. The conversation scenario was as follows: (1) greeting, (2) engaging in small talk, (3) introducing the purpose of the conversation (i.e., discussing participants’ interpersonal stress event) and guaranteeing the confidentiality of the conversation, (4) asking several questions about participants’ interpersonal stress events and (only in the informational or emotional support conditions) providing informational or emotional support, respectively, (5) asking whether participants would change their behavior and the extent to which they felt their negative emotions changed, and (6) concluding the conversation.

At the end of the conversation, the chatbot sent a link to a survey. In the survey, participants’ perceived effectiveness of the chatbot’s messages and mind perception for the chatbot were measured. Implicit mind perception was assessed following the measurement of explicit mind perception. This sequence was intended to mitigate the potential influence of exposure to the scenarios of implicit mind perception measurement, which could have affected responses to the items assessing explicit mind perception. Finally, after collecting the participants’ demographic information, the study was concluded.

## 4 Results

### 4.1 Dividing data into implicit/explicit mind perception groups

Before conducting hypothesis testing, we preprocessed the implicit and explicit mind perception data. For implicit mind perception, we classified the data into *implicit mind perception* group (*n* = 83) if the score on the measurement was 1, and *no implicit mind perception* group (*n* = 54) if the score was 0. For explicit mind perception, we also divided the data into two groups (*explicit mind perception* and *no explicit mind perception*) using the mean total score of the scale. If the mean score of explicit mind perception was greater than 3, the data were assigned to the explicit mind perception group (*n* = 82); otherwise, they were assigned to the no explicit mind perception group (*n* = 55).

We used the total score of explicit mind perception, rather than individual scores for agency and experience, to maintain equivalence between explicit and implicit mind perceptions since we only measured the overall implicit one. Then, we set the mean total score of 3 as the cut-off point. Unless the participants explicitly denied statements describing that the chatbot had a human-like mind, we interpreted that some degree of mind attribution occurred. In other words, if the participants rated at least one item 4 or higher (*neutral, slightly agree, moderately agree, strongly agree*), resulting in mean scores greater than 3, we considered them perceiving a human-like mind.

### 4.2 Mind perception and perceived message effectiveness

We conducted t-tests to examine whether the degree of perceived message effectiveness differed depending on participants’ explicit and implicit mind perception, respectively. The results revealed that the difference between the implicit mind perception and no implicit mind perception groups was statistically significant (*t* (135) = 2.84, *p* = 0.005, Cohen’s *d* = 0.50). The implicit mind perception group (*M* = 4.6, SD = 1.2) perceived the chatbot messages as more effective than the no implicit mind perception group (*M* = 4.0, SD = 1.3). The difference between the explicit mind perception and no explicit mind perception groups was also statistically different (*t* (134.89) = 6.78, *p* < 0.001, Cohen’s *d* = 1.09). The explicit mind perception group (*M* = 5.1, SD = 0.9) also showed higher score in perceived message effectiveness than the no explicit mind perception group (*M* = 3.9, SD = 1.3).

### 4.3 Mind perception and different social support

We explored whether the relation between mind perception and perceived message effectiveness varied depending on the type of social support; in other words, whether the association was stronger when the chatbot provided emotional support. The means and standard deviations of perceived message effectiveness for each condition are shown in Table 1.

**Table 1:** Means and standard deviations of perceived message effectiveness.

	*M*	SD
**Base Condition**		
No Implicit Mind Perception	4.6	1.2
Implicit Mind Perception	4.5	1.2
No Explicit Mind Perception	4.1	1.3
Explicit Mind Perception	5.2	0.7
**Informational Support Condition**		
No Implicit Mind Perception	4.2	1.0
Implicit Mind Perception	4.8	1.3
No Explicit Mind Perception	4.2	1.3
Explicit Mind Perception	5.2	0.9
**Emotional Support Condition**		
No Implicit Mind Perception	3.2	1.3
Implicit Mind Perception	4.5	1.1
No Explicit Mind Perception	3.4	1.3
Explicit Mind Perception	4.9	0.9

#### 4.3.1 Examining a potential confounding factor: chatbot’s message content

In the informational support condition, the chatbot sent different advice depending on the type of stress event (i.e., relationship building, maintaining, and ending) to make conversations more natural and encourage participants’ engagement. To check the potential confounding effect of different message content, we conducted a one-way ANOVA test to examine whether different types of advice had an effect on perceived message effectiveness. The results revealed that perceived message effectiveness remained consistent across different types of advice (*F* (2, 45) = 0.00, *p* = 0.999, η^2^ = 0.00). The means and standard deviations of perceived message effectiveness for each type of advice were as follows: relationship building, *M* = 4.6, SD = 1.3; relationship maintaining, *M* = 4.6, SD = 1.2; and relationship ending, *M* = 4.6, SD = 1.4.

#### 4.3.2 Informational support

We conducted two-way ANOVA tests to examine whether the effects of providing informational support on perceived message effectiveness varied depending on the participants’ implicit or explicit mind perception, respectively. The results revealed that the interaction effect between informational support and implicit mind perception was not statistically significant (*F* (1, 86) = 1.26, *p* = 0.264, partial η^2^ = 0.01). The degree of perceived message effectiveness was not significantly different between the base and the informational support conditions (*F* (1, 86) = 0.76, *p* = 0.387, partial η^2^ = 0.01). Additionally, the difference between the implicit mind perception and the no implicit mind perception groups was not statistically significant (*F* (1, 86) = 0.01, *p* = 0.926, partial η^2^ = 0.00). For explicit mind perception, the interaction effect between informational support and explicit mind perception was also not statistically significant (*F* (1, 86) = 0.19, *p* = 0.666, partial η^2^ = 0.00). The degree of perceived message effectiveness was not significantly different between the base and the informational support conditions (*F* (1, 86) = 0.26, *p* = 0.615, partial η^2^ = 0.00). However, the explicit mind perception group perceived the chatbot’s messages as more effective than the no explicit mind perception group (*F* (1, 86) = 11.69, *p* < 0.001, partial η^2^ = 0.12).

#### 4.3.3 Emotional support

**Figure 3:**
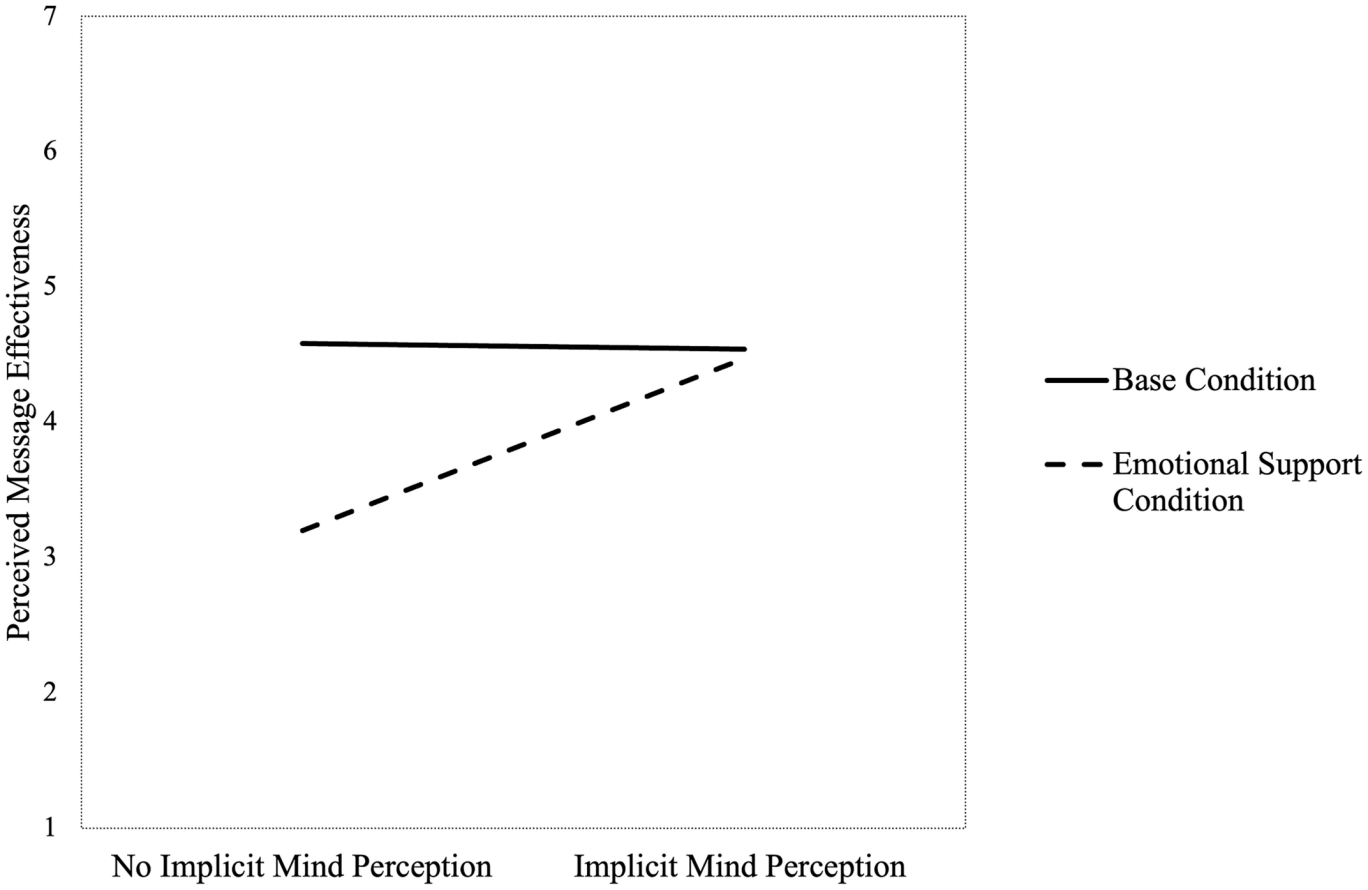
Interaction effect between providing emotional support and implicit mind perception.

We conducted two-way ANOVA tests to examine whether the effects of providing emotional support on perceived message effectiveness varied depending on the participants’ implicit or explicit mind perception. The interaction effect between emotional support and implicit mind perception was statistically significant (*F* (1, 85) = 6.31, *p* = 0.014, partial η^2^ = 0.07). The difference in perceived message effectiveness between the implicit mind perception and the no implicit mind perception groups was more pronounced in the emotional support condition compared to the base condition, as shown in Figure 3. Perceived message effectiveness was lower in the emotional support condition compared to the base condition (*F* (1, 85) = 12.27, *p* < 0.001, partial η^2^ = 0.13). In contrast, there was no significant difference in perceived message effectiveness between the implicit mind perception group and the no implicit mind perception group (*F* (1, 85) = 0.01, *p* = 0.925, partial η^2^ = 0.00). For explicit mind perception, the result showed that the interaction effect between emotional support and explicit mind perception was not statistically significant (*F* (1, 85) = 0.53, *p* = 0.468, partial η^2^ = 0.01). However, the extent to which participants perceived the chatbot’s messages as effective was lower in the emotional support condition compared to the base condition (*F* (1, 85) = 5.01, *p* = 0.028, partial η^2^ = 0.06). In addition, the explicit mind perception group considered the chatbot’s messages as more effective than the no explicit mind perception group (*F* (1, 85) = 11.84, *p* < 0.001, partial η^2^ = 0.12).

## 5 Discussion

This study explored whether the degree to which users perceived the chatbots’ message as effective differed depending on the perception of chatbots’ minds and whether the strength of the relationship between them varied depending on the type of support provided by the chatbots. The *t*-test results showed that implicit and explicit mind perception in a chatbot was related to increases in perceived message effectiveness. Furthermore, the two-way ANOVA results revealed that, for explicit mind perception, positive relationship was still significant after considering the effects of support type. In contrast, the relationship between implicit mind perception and perceived message effectiveness was not significant after considering the effects of support type. These results suggest that the relationship between implicit mind perception and perceived message effectiveness was influenced by the type of social support. Thus, Hypothesis 1-2 was supported while Hypothesis 1-1 was only partially supported.

According to the two-way ANOVA results, there was a significant interaction effect between implicit mind perception and support type on perceived message effectiveness only when the chatbot provided emotional support. However, there was no significant interaction effect between explicit mind perception and support type for both informational and emotional support. In sum, only when participants did not implicitly attribute a human-like mind to the chatbot, providing emotional support (but not informational support) decreased the extent to which the participants considered the chatbots’ messages as effective. Thus, only Hypothesis 2-1 was supported. Additionally, the two-way ANOVA results revealed that overall, the degree of perceived message effectiveness did not vary between the base and informational support conditions, while perceived message effectiveness was lower in the emotional support condition compared to the base condition.

This study was the first to directly measure user mind perception and investigate how it was associated with the extent to which users perceived the chatbot message as effective. Our study was particularly pioneering in separately investigating implicit and explicit mind perception in the context of exploring chatbot support. As we measured implicit mind perception after the explicit one, exposure to the explicit mind perception scale might have primed mind attribution when the participants answered on the implicit one. However, nearly 40% of participants did not implicitly attribute a human-like mind even after the exposure, suggesting that the potential priming effect might not be significant. Therefore, the distinct examination of implicit and explicit mind perception was possible.

Our study contributes significantly to understanding how perceiving human-like minds in chatbots is associated with the user experience for chatbots’ social support. First, our work demonstrated the importance of explicit mind perception in inducing positive users’ reactions to the chatbots’ social support, regardless of the type of support provided by the chatbots. As suggested by Lee et al. (2020), when users explicitly perceive chatbots as mindful entities, interactions with chatbots might be considered as more meaningful, consequently fostering a more favorable reception of the chatbots’ social support. Mindfully experiencing the chatbot’s humanness might encourage the participants to perceive the conversation with the chatbot as more valuable.

Second, our findings revealed that the importance of implicit mind perception in positive user experience differed depending on the type of support provided by chatbots. Specifically, implicit mind perception is more important when chatbots provide emotional support, as emotional support implies that the support provider has the ability to understand and feel emotions. To reiterate, users appreciate chatbots’ social support only when they perceive the chatbots as having the capacity to provide such support at least implicitly. In general, people consider chatbots as having agency to some extent, but lacking experience (Gray et al., 2007; Jacobs et al., 2022). Thus, even though users do not implicitly perceive human-like minds in chatbots, they appreciate the chatbots’ informational support because chatbots are considered to have agency, which is required to provide informational support. In contrast, since chatbots are regarded as lacking experience, their emotional support causes discomfort if users do not humanize and attribute the experience to them. Gray and Wegner (2012) revealed that perceiving experience in a machine led to eeriness and unease from users as machines are considered to lack experience. Relatedly, users who did not appreciate an AI agent’s empathic expression felt that it pretended to understand their inner states (Urakami et al., 2019). On the contrary, users who appreciated chatbots’ empathic expressions reported feeling as if they were talking to a human (Fitzpatrick et al., 2017; Bae Brandtzæg et al., 2021). Thus, implicitly perceiving chatbots as having the capacity to deliver a certain type of support might be necessary to induce the desired reactions from users.

Third, simply encouraging users to write down their stress events by asking several questions (what the chatbot performed in the base condition of the experiment) has a similar or potentially higher positive impact on the perceived effectiveness of the message compared to providing additional informational or emotional support. Our findings are consistent with the previous research, which suggests that writing about stressful experiences is beneficial since it can facilitate introspection and self-evaluation (Ta et al., 2020; Bae Brandtzæg et al., 2021). These results implicate that users might expect different forms of support from chatbots rather than mimicking humans’ support. Some researchers suggested that, because people nowadays know various AI agents, understand their unique characteristics, and have experience with them, they have different expectations from them and, further, interact with them differently to how they interact with humans (Gambino et al., 2020). For example, users expect and recognize AI agents to be non-judgmental because they are essentially machines, encouraging users to tell their innermost stories without fear, unlike when conversing with other humans (Ta et al., 2020). Moreover, users recognize that AI agents lack emotions and expect them to listen and react to their stories without becoming tired (Kim et al., 2018). Another possible explanation for the results is the insufficient effects of the informational and emotional support. The informational support provided by our chatbot included advice that was general rather than customized to participants’ exact situations. In addition, the chatbot mostly retrieved typical and general empathic expressions (e.g., “I would feel the same way as you.”) for emotional support. The generality and typicality of the provided support might have reduced its effects.

Lastly, our work has some practical implications. Specifically, the results encourage practitioners to design chatbots’ social support differently depending on whether users implicitly perceive human-like minds in the chatbots or not. Informational support is more dependable in eliciting desired effects regardless of users’ implicit mind perception, whereas emotional support is not. If users implicitly perceive human-like minds in chatbots, they may expect the same form of support that humans would provide. In other words, users may expect the chatbots to understand and empathize with their experience and respond to them with appropriate emotional reactions. However, if users do not consider chatbots as mindful entities, even in implicit ways, they may not want the agents to pretend to understand their inner experience. Other forms of support that computers can perform (e.g., listening to users’ stories without getting bored or offering useful information) might be more appreciated by users.

## 6 Limitations and future research

The present study has some limitations. First, we did not manipulate the degree of participants’ mind perception but measured it. Consequently, we could not investigate the casual effects of mind perception on users’ attitudes toward the support received. We decided to measure users’ mind perception rather than manipulate it because one’s tendency to anthropomorphize and attribute humanness is influenced by various factors (Epley et al., 2007; Waytz et al., 2010), except for chatbots’ human-likeness, and therefore, hard to be perfectly manipulated. Nevertheless, to investigate the causal effects of mind perception, future research should explore ways to manipulate users’ mind perception as perfectly as possible.

Second, we used only one task for assessing implicit mind perception because it was the only task that was determined to be appropriate to use in the context of human-chatbot interactions (i.e., the white lie scenario). Future research should develop validated measures to assess implicit mind perception in chatbots and conduct a more in-depth investigation.

Third, our chatbot sometimes predicted participants’ emotions and situations incorrectly. Prediction failure may impair the positive user perception of social support. To accurately examine user experience of social support, future studies should conduct experiments with Wizard-of-Oz methods (Dahlbäck et al., 1993) or more sophisticated prediction models such as the large language models.

Finally, the current study was slightly underpowered to detect medium effects in the ANOVA tests. It might be accompanied by the reduced sample and small effects of chatbot support caused by a one-time, short interaction. Previous studies that are consistent with our main results (e.g., Fitzpatrick et al., 2017; Urakami et al., 2019; Ta et al., 2020; Bae Brandtzæg et al., 2021), however, support the significance of our findings. Nevertheless, future studies should be conducted with larger samples and more effective chatbot support to validate the replication of our results.

## 7 Conclusion

Our study shows that user mind perception and the properties of different social support should be considered together to offer more efficient chatbot support. With the rapid advance of AI technologies, chatbots are increasingly permeating people’s daily lives and are utilized to enhance individuals’ psychological well-being by providing various social support. Thus, in this situation, it is necessary to explore ways to improve the quality of user experience with chatbot support. Our study suggests one way to enhance user acceptance of chatbot support, which is to provide different types of social support (e.g., helpful advice or empathy for users’ situations) in consideration of the users’ mind perception.

## Data availability statement

The datasets presented in this article are not readily available because the conversation data should be confidential, and some participants did not consent to their data being available to other researchers. Requests to access the datasets should be directed to IL, ijlee37@snu.ac.kr.

## Ethics statement

The studies involving humans were approved by Seoul National University institutional review board. The studies were conducted in accordance with the local legislation and institutional requirements. The institutional review board waived the requirement of written informed consent for participation from the participants because the study was conducted online. Online consent was obtained instead of written consent.

## Author contributions

IL: Writing – original draft, Conceptualization, Formal analysis, Funding acquisition, Investigation, Methodology. SH: Supervision, Writing – review & editing.

## Footnotes

^1^chatfuel.com

^2^dialogflow.cloud.google.com
